# VO_2_FITTING: A Free and Open-Source Software for Modelling Oxygen Uptake Kinetics in Swimming and other Exercise Modalities

**DOI:** 10.3390/sports7020031

**Published:** 2019-01-24

**Authors:** Rodrigo Zacca, Rui Azevedo, Pedro Figueiredo, João Paulo Vilas-Boas, Flávio A. de S. Castro, David B. Pyne, Ricardo J. Fernandes

**Affiliations:** 1 Centre of Research, Education, Innovation and Intervention in Sport, Faculty of Sport, University of Porto, Porto 4200-450, Portugal; jpvb@fade.up.pt (J.P.V.-B.); ricfer@fade.up.pt (R.J.F.); 2 Porto Biomechanics Laboratory (LABIOMEP), University of Porto, Porto 4200-450, Portugal; 3 CAPES Foundation, Ministry of Education of Brazil, Brasília 70040-031, Brazil; 4 CESPU, Institute of Research and Advanced Training in Health Sciences and Technologies (IINFACTS), Gandra PRD 4585-116, Portugal; rui.azevedo@iucs.cespu.pt; 5 Portugal Football School, Portuguese Football Federation, Cruz Quebrada-Dafundo 1495-433, Portugal; pedfig@me.com; 6 Aquatic Sports Research Group, Universidade Federal do Rio Grande do Sul, Porto Alegre 90690-200, Brazil; souza.castro@ufrgs.br; 7 Research Institute for Sport and Exercise, University of Canberra, Canberra, ACT 2617, Australia; david.pyne@canberra.edu.au

**Keywords:** exercise, software, free, open-source, oxygen uptake kinetics, modelling

## Abstract

The assessment of oxygen uptake (VO_2_) kinetics is a valuable non-invasive way to evaluate cardiorespiratory and metabolic response to exercise. The aim of the study was to develop, describe and evaluate an online VO_2_ fitting tool (VO_2_FITTING) for dynamically editing, processing, filtering and modelling VO_2_ responses to exercise. VO_2_FITTING was developed in Shiny, a web application framework for R language. Validation VO_2_ datasets with both noisy and non-noisy data were developed and applied to widely-used models (n = 7) for describing different intensity transitions to verify concurrent validity. Subsequently, we then conducted an experiment with age-group swimmers as an example, illustrating how VO_2_FITTING can be used to model VO_2_ kinetics. Perfect fits were observed, and parameter estimates perfectly matched the known inputted values for all available models (standard error = 0; p < 0.001). The VO_2_FITTING is a valid, free and open-source software for characterizing VO_2_ kinetics in exercise, which was developed to help the research and performance analysis communities.

## 1. Introduction

Successful sporting performance is the result of a complex interaction between many factors, which often involves testing procedures to evaluate the effects of training programs [1]. From a diversity of tests, variables and prediction models [2,3,4], sports scientists characterize dynamic profiles (kinetics) of cardiorespiratory variables to better understand the control mechanisms of muscle energetics and oxidative metabolism [4]. These profiles can inform the preparation of the annual training plan, periodization of training mesocycles and microcycles and the prescription of individual training sets [5]. However, for the effective application of cardiorespiratory variables, a detailed understanding of the basic principles of oxygen uptake (VO_2_) kinetics is required. The rate at which VO_2_ responds to metabolic demand changes at the onset of exercise is dependent on the capacity of the cardiorespiratory and muscular systems to react appropriately [6]. Understanding VO_2_ kinetics involves quantifying physiological mechanisms responsible for the dynamic VO_2_ response to exercise (on-transient kinetics) and its subsequent recovery (off-transient kinetics) [4]. Although the mechanisms that control the regulation of the O_2_ transport/utilization system to changes in metabolic demand have been studied over decades, quantifying VO_2_ kinetics has gained popularity during the last decade [4]. In fact, some VO_2_ kinetic parameters have different and eventually faster and higher responsiveness to training stimuli than maximal oxygen uptake (VO_2max_) [7,8,9].

VO_2_ assessment in exercise, followed by VO_2_ profile mathematical modelling, is a useful non-invasive method for studying muscle oxidative metabolism. The VO_2_ response following the onset of a specific intensity can be defined by the underlying exercise-intensity domain (moderate, heavy, severe and extreme) [10,11,12]. At the moderate intensity domain (i.e., below and at the anaerobic threshold), VO_2_ begins to increase within the first breath (Phase I or cardiodynamic component), and after a brief time delay (~15–20 s), there is an exponential increase of VO_2_ (Phase II, fundamental or primary component, to achieve a subsequent steady state (Phase III) [13,14,15]. At the heavy intensity domain, after the cardiodynamic phase, VO_2_ continues rising before a secondary VO_2_ elevation becomes apparent after ~90–120 s (the slow component of VO_2_ kinetics, VO_2sc_). This increase is combined with a faster primary response until a delayed steady state is attained, exhaustion ensues or exercise ends [13,16,17]. At even higher work rates, at the severe intensity domain, VO_2_ cannot be stabilized, rising rapidly and exponentially to VO_2max_ [15,18]. At this intensity domain, VO_2sc_ is more pronounced compared to heavy exertion, rising inexorably until fatigue ensues [19]. Finally, in the extreme domain, the work rate is so intense that the task finishes before the VO_2max_ can be achieved [4,20]. At this intensity, VO_2_ is characterized by the development of a fast component with insufficient time for a VO_2sc_ to appear [21].

Analysis of VO_2_ kinetics provides useful information about the rate of adjustment of oxidative phosphorylation, which permits separate assessment of the relative contribution of the energy systems delivery, substrate utilization and the time endured during exercise [21]. Higher exercise tolerance is essentially associated with changes in the fast and slow components of the VO_2_ kinetics, particularly the time constant of the primary component (τ_p_) and the amplitude of the slow component (A_sc_), which are relevant for performance analysis [21,22]. Children also exhibit a slow-component response to exercise, which is consistent with an age-dependent change in muscles’ potential for O_2_ consumption. The inherent increase in the signal-to-noise ratio from childhood through adulthood might mask any clear changes in ventilatory variables [23]. A smaller value for τ_p_ results in faster attainment of steady state, while a delayed or more slowly developing A_sc_ is associated with enhanced exercise tolerance [4].

Given that rapid feedback from testing is necessary for coaches to better plan training sessions, tools for analysing the VO_2_ response (data editing, processing, filtering and modelling) should be available, effective and relatively straightforward. However, free and open-source software supported by these features is not yet available. Thus, end-users usually need to develop customized in-house tools, which require mastery of complex mathematical modelling, as well as basic knowledge of respiratory physiology. Therefore, the aim of the study was two-fold: (i) to develop, describe and evaluate a VO_2_ fitting tool for dynamically editing, processing, filtering and modelling VO_2_ responses to exercise; and (ii) to verify, using this VO_2_ fitting tool, the goodness of fit between different models and respective confidence intervals of VO_2_ kinetics parameters obtained from one swimming event to illustrate some of the software capabilities. 

## 2. Materials and Methods

We developed a software package (VO_2_FITTING) and conducted an experiment illustrating how it can be used to edit, process, filter and model VO_2_ responses in exercise. To this end, we chose swimming as an example, but other examples are available as supporting information (S1 File), illustrating selected options on VO_2_FITTING, which should be useful for research and performance analysis in sports. We used raw data from a pool-based fixed-distance even-paced swim test (400-m swim test, T400) performed at the severe intensity domain [24], without any constraint for parameter estimates (see “Constraining Parameters in Curve Fitting” in S1 File). We chose the severe intensity domain as an example since it is expected that there is a lower VO_2_ signal-to-noise ratio than in heavy and moderate domains.

### 2.1. Development and Validation of VO_2_FITTING Software: Source Code, Requirements, Availability and License

VO_2_FITTING offers functionalities that confer enough flexibility to compare simultaneously several respiratory responses with sufficient precision to meet researcher and performance analyst requirements (S1 File). VO_2_FITTING runs online, inside a browser, requiring a Shiny server, which can be configured for local or shared access by multiple users, and importantly does not require an internet connection while modelling VO_2_ kinetics (S1 File). News about the application, source code, installation instructions and other documentation can be verified on the landing page (https://shiny.cespu.pt/vo2_news/). The latest version of VO_2_FITTING will be permanently available in the repository. The source code of VO_2_FITTING is released under a GNU General Public License version 3 (GPL-3; https://www.r-project.org/Licenses/GPL-3). Software packages which are covered by this license are free and open-source, even after each new release. This license ensures that everyone can use, modify and contribute to the software. Validation VO_2_ datasets were developed and applied to 7 widely-used models for describing different intensity transitions (3 mono-exponential, 2 bi-exponential, 1 tri-exponential and 1 logistic model; see S1 File for details).

### 2.2. Subjects (Swimming Experiment)

Twenty age-group swimmers (10 males and 10 females) volunteered to participate in this study. Their main physical and training frequency characteristics were: 14.9 ± 0.9 and 14.2 ± 0.9 years old, body mass 67.2 ± 3.6 and 52.7 ± 6.9 kg, height 170.8 ± 2.6 and 160.0 ± 6.3 cm, arm span 174.5 ± 8.1 and 164.1 ± 10.5 cm (mean ± SD for male and female swimmers, respectively), 6–7 swimming sessions and 2–3 h of dryland training per week in the same squad and under direction of the same coach, ≥ five years of competitive experience and 538 ± 73 *Fédération Internationale de Natation* (FINA) points (year: 2015) from the main event during competition (https://www.swimrankings.net/). The most individual representative stroke was freestyle for 17 swimmers, backstroke for 2 swimmers and breaststroke for 1 swimmer. Pubertal maturation stage [25] was similar for both males and females (late pubertal to post pubertal). All subjects were informed about the benefits and risks of participating in the investigation prior to signing an institutionally approved informed consent form. In addition, each swimmer’s parents or guardian provided written consent prior to their participation. The study was approved by the ethics board (Process CEFADE 04.2017) of the Faculty of Sport of University of Porto and performed according to the Helsinki Declaration.

### 2.3. Experimental Methodology

Prior to the experiment, swimmers were familiarized for three months, 2–3 times per week, with a snorkel and nose clip. The experimental protocol took place in a 25-m indoor pool (water (~27 °C) and air (~25 °C) temperature, and ~65% relative humidity). Swimmers were tested at the same time of the day and instructed not to perform strenuous exercise on the day before. Swimmers were instructed to follow their normal diet in the day preceding the testing, and to have a light meal (breakfast) 3 h before, including ~500 mL of water or a beverage but no caffeine. Following a randomized order, each swimmer performed ~800-m front crawl warm up at a moderate intensity, and soon after, a T400. The T400 is commonly used to assess aerobic power in swimmers, given its pace is situated on the severe intensity domain [16]. Although the breathing snorkel used for respiratory gas collection does not add additional hydrodynamic drag [26], in-water starts and open turns (without underwater gliding) were used given the inherent physical restrictions of the snorkel. Subsequently, the VO_2_FITTING software was applied (see S1 File) for editing, processing and modelling VO_2_ response from each swimmer. Although seven different models are available to describe different intensity transitions (see S1 File for details), the goodness of fit of two different models (mono- and bi-exponential) and respective confidence of VO_2_ kinetics parameters were verified during this experiment with swimmers to check if the current data are consistent with the expected behaviour for the exercise response in the severe intensity domain.

### 2.4. Experimental Measurements

Respiratory and pulmonary gas-exchange data were measured breath-by-breath using a low hydrodynamic resistance respiratory snorkel and valve system (AquaTrainer^®^, Cosmed, Rome, Italy) [27]. The AquaTrainer^®^ was connected to a telemetric portable gas analyzer (K4b^2^, Cosmed, Rome, Italy) and suspended at a height of 2 m over the water in a steel cable (designed to minimize disturbance of the normal swimming movements) [26]. The telemetric portable gas analyzer and turbine volume transducer were calibrated (following the manufacturer instructions) before each testing session with gases of known concentrations (16% O_2_ and 5% CO_2_) and 3 L syringe, respectively.

### 2.5. Models and Parameters Estimation

To increase the VO_2_ signal-to-noise ratio, 2–3 exercise transitions are usually performed per participant [28,29,30,31]. These are time-aligned and ensemble averaged to yield a single profile per participant [32,33,34,35]. This feature is available in the VO_2_FITTING, of which an example is shown (S1 File). However, it was not used in the current experiment, since even only two repetitions by each swimmer are not easily measured poolside. In fact, squads are often large and facilities, equipment and sports science expertise hard to come by. Thus, instead of identical repetitions, a single test is commonly used. Therefore, we employed an experimental model where each swimmer (n = 20) performed a single 400-m front crawl even-paced swim test (T400), in randomized order, same warm-up, arousal, time of day and time of testing within the training period.

The VO_2_ kinetics parameters were estimated, including the precision of estimation (confidence limits), by bootstrapping [36,37,38] (see Statistical Analysis section for more details). Parameter estimates and the goodness of fit of each model (mono- and bi-exponential) were only analyzed with raw data.

The first 20 s of data after the onset of exercise (cardiodynamic phase) were not considered for VO_2_ kinetics analysis [39]. For each swimmer, the on-transient was modelled with mono- and bi-exponential models (Equations (1) and (2)), characterizing the exercise VO_2_ response during the T400:

(1) $$VO_{2}\left(t\right)=A_{0}+H\left({t-\mathrm{TD}}_{p}\right)\times A_{P}\left({1-e}^{{-(t-\mathrm{TD}}_{p}{)/\tau }_{p}}\right)$$

(2) $$VO_{2}\left(t\right)=A_{0}+H\left({t-\mathrm{TD}}_{p}\right)\times A_{P}\left({1-e}^{{-(t-\mathrm{TD}}_{p}{)/\tau }_{p}}\right)+H\left({t-\mathrm{TD}}_{\mathrm{SC}}\right)\times A_{\mathrm{sc}}\left({1-e}^{{-(t-\mathrm{TD}}_{\mathrm{sc}}{)/\tau }_{\mathrm{sc}}}\right)$$

where VO_2_(t) (mL·kg^−1^·min^−1^) represents the VO_2_ normalized to body mass at the time t, A_0_ is the VO_2_ at rest (2 min average; mL·kg^−1^·min^−1^). A_p_ and A_sc_ (mL·kg^−1^·min^−1^), TD_p_ and TD_sc_ (s), and τ_p_ and τ_sc_ (s) are respectively the amplitudes, the corresponding time delays and time constants of the fast and slow VO_2_ components. H represents the Heaviside step function (Equation (3)) [40]:

(3) $$H\left(t\right)=\{\begin{array}{c} 0,t<0\\ 1,t\geq 0 \end{array}$$

VO_2_ at the end was calculated as the average of the last 60 s of exercise for both models. Since the asymptotic value of the second function is not necessarily reached at the end of the exercise, the amplitude of the A_sc_ at the end of the T400 (A_sc_end_) was also calculated (Equation (4)) (see “Auxiliary Reports” in the S1 File) [22,41]:

(4) $$A_{\mathrm{sc}\_\mathrm{end}}=A_{\mathrm{sc}}({1-e}^{{-(t}_{\mathrm{end}}{-\mathrm{TD}}_{\mathrm{sc}}{)/\tau }_{\mathrm{sc}}})$$

where t_end_ is the time at the end of the T400. The A_sc_ represented the difference between the VO_2_ at the end (average of the last 60 s) and “A_p_ + A_0_” was also calculated using the available option from VO_2_FITTING.

The R (R Core Team, 2015), a free software environment for statistical computing and graphics, was used to perform all the computations in the study, with the support of the Shiny package [42] (https://cran.r-project.org/web/packages/shiny/index.html) and other dependencies (see S1 File). Notable dependencies are *minpack.lm* and *nlstools*, PACKAGES for non-linear least-square regression analysis [43,44], used to fit he VO_2_ response from the single T400 for both mono-and bi-exponential functions.

### 2.6. Statistical Analysis

Noisy (Gaussian) and non-noisy datasets were developed for seven different models to describe different intensity transitions (3 mono-exponential, 2 bi-exponential, 1 tri-exponential and 1 logistic model; see S1 File for details), both are provided as downloadable spreadsheets in the supporting information (S2 File). Subsequently, VO_2_ data outputs as a function of time were created through these files and uploaded in the application, verifying whether the fitted parameters perfectly matched the known input values. Moreover, all these spreadsheets (S2 File) can be employed by the end-user to generate different datasets, even with different ranges of input values and suitable for specific scenarios, to verify and validate the software response. We used bootstrapping with 1000 samples (with replacement from the observed residuals), adjustable in the interface, to estimate parameters of mono- and bi-exponential fitting models for each swimmer T400 [36,37,38]. The mean, standard deviation, coefficient of variation, and 95% confidence intervals were calculated for each parameter estimate (bootstrapping analysis is available in VO_2_FITTING; see “Output Options” in the S1 File). To verify the goodness-of-fit for both mono- and bi-exponential models, Shapiro–Wilk (residuals distribution) and ANOVA F-test (with respective residuals sum of the squared from the differences between both models) were applied. A level of significance of 0.05 was used in all tests.

## 3. Results

**Figure 1:**
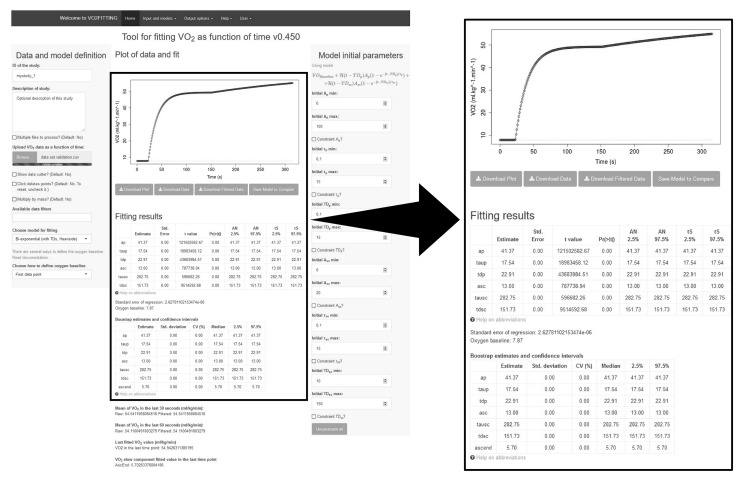
An example of (bi-exponential) validation dataset uploaded from VO_2_FITTING (middle). Upper-left and right corners show *data and model definition* menu, and the *model initial parameters* menu (without any constraint for parameter estimates).

VO_2_ output data as a function of time obtained from the validation datasets (S2 File) generated perfect fits. Moreover, the parameter estimates perfectly matched the known inputted values for all seven available models (standard error = 0; p < 0.001). An example of a bi-exponential model validation dataset uploaded in VO_2_FITTING is displayed in Figure 1, with parameter estimates perfectly matching the known inputted values.

Table 1 shows an overview of all parameter estimates for each model, particularly the mean parameter estimates for all swimmers (fitted individually), standard deviation, 95% confidence interval and mean coefficient of variation. The mean swimming performance in T400 was 5:15 ± 0:20 min:s (males: 5:04 ± 0:06 min:s; females; 5:27 ± 0:17 min:s).

**Figure 2:**
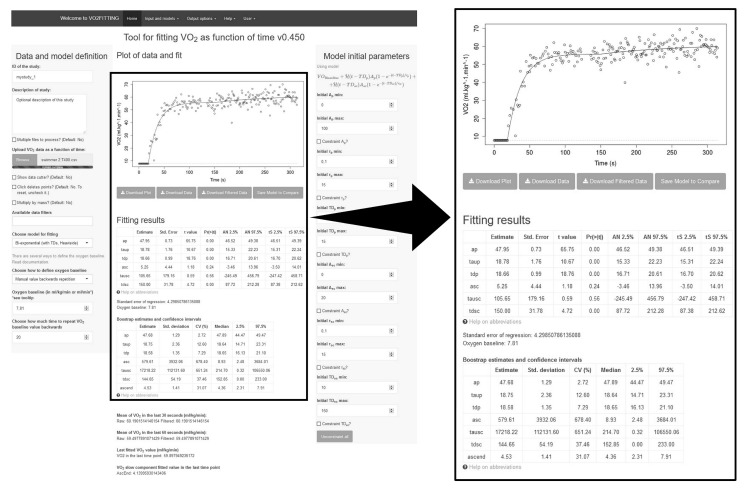
VO_2_FITTING home menu with an individual example of a T400 modelled VO_2_ response (bi-exponential) in the middle. *Data and model definition*, and *model initial parameters* menu without any constraint for parameter estimates are presented in upper-left and right corner, respectively.

Figure 2 shows the VO_2_FITTING home menu with an example of a T400 modelled VO_2_ response (bi-exponential). Software options include joining multiple observations, data filtering, specific data point deletions, multiple options related to A_0_, several fitting models, output reports, and constraint fitting ranges for parameters when the fitting fails with default values. A screenshot from the bottom of the VO_2_FITTING home menu detailing residuals plots to evaluate the goodness of fit of the T400 modelled VO_2_ response (bi-exponential) of the same swimmer are presented in the supporting information (S1 File–Figure 2).

**Figure 3:**
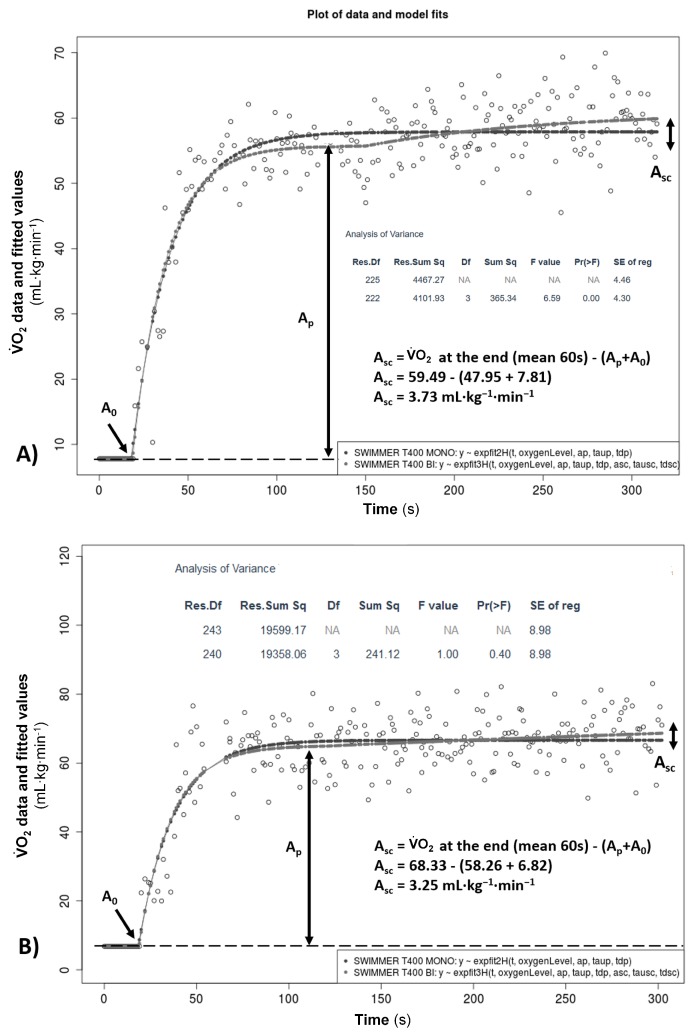
Examples of goodness-of-fit analysis between mono- and bi-exponential models of a typical T400 VO_2_ response from two swimmers. (**A**) bi-exponential model being superior; (**B**) No difference between mono- and bi-exponential models. Shapiro–Wilk (residuals distribution) and ANOVA F-test (with respective residuals sum of the squared from the differences between both models) detailed on the top of both graphics. * Res.Df: residual degree of freedom; Res.Sum Sq: residual sum of square; Df: degree of freedom; Sum Sq: sum of square; F value: for testing the hypothesis that the group means for that effect are equal; Pr(>F): the significance probability value associated with the F Value; SE of reg: standard error of regression.

Figure 3 shows two examples of mono- versus bi-exponential fits comparisons from a typical T400 VO_2_ response (with corresponding residual plots for each model). On the top of Figure 3, the F-test indicated that the bi-exponential model was superior for this swimmer. Likewise, the bi-exponential model best fitted for 15 swimmers, presenting a smaller residual sum of squares and standard error of regression (p < 0.05). The mono-exponential model was not superior for any swimmer since the F-test did not show differences between models for the remaining five swimmers, as illustrated on the bottom of Figure 3. The A_sc_ calculated as the difference between the VO_2_ at the end (average of the last 60 s) and “A_p_ + A_0_” was 6.3 ± 2.7 mL·kg^−1^·min^−1^. Model comparison was similar between gender, since the bi-exponential model best fitted for 70 and 80% of males and females, respectively.

We also added an example in the supporting information (S1 File), in which a comparison between swimming and running was performed at the same relative intensity. We observed that the coefficients of variations from parameter estimates were predominantly higher in swimming than running, thus illustrating some of the available options of VO_2_FITTING, useful for research and performance analysis in elite, sub-elite or recreational athletes.

## 4. Discussion

Performance analysts typically take several hours per week to provide objective information for athletes and coaches, helping them to understand and improve performance in sports. The use of technology and software by these professionals is crucial. Analysts need to give rapid performance diagnosis, since coaches need immediate feedback for planning, modifying and evaluating training sessions. To this end, dynamic and feasible fitting tools for VO_2_ kinetics analysis should be available. In this study, VO_2_ data outputs were uploaded from noisy and non-noisy datasets to assess the concurrent validity of available models from VO_2_FITTING, and some experiments were performed to illustrate its applicability.

Analysis of VO_2_ kinetics enables a non-invasive assessment of the effectiveness of training programs, providing relevant information about exercise tolerance determinants. Commercial software requires mastery of complex software for mathematical modelling (e.g., Matlab, Mathworks, Natick, USA, www.mathworks.com; LabVIEW™, National Instruments, Austin, TX, USA, http://www.ni.com/en-us/shop/labview.html), and basic knowledge of respiratory physiology for research and performance analysts in sports [14,35,45]. The VO_2_FITTING software solves that constraint, allowing straightforward analysis of VO_2_ kinetics in exercise with a feasible graphical interface. Although some of the available commercial software also provides the end-user with relatively straightforward options for VO_2_ kinetics data analysis (e.g., Origin, OriginLab, Northampton, MA, USA, www.originlab.com; GraphPad Prism, GraphPad Software, San Diego, CA, USA, https://www.graphpad.com/; SigmaPlot, Systat Software, San Jose, CA; www.sigmaplot.co.uk), VO_2_FITTING goes further since it is free and open-source with built-in features that are commonly used in VO_2_ kinetics modelling, which appears to have promise as a useful tool for the research and performance analysis communities.

Every VO_2_ breath-by-breath signal has non-uniformities in the underlying breathing pattern [32], which are relevant for its variability, particularly if corrections for the differences between alveolar and mouth O_2_ exchanges are not taken into consideration [46]. We chose swimming as an example, using raw data from a pool-based fixed-distance even-paced swim test performed at the severe intensity domain, since it is expected that there is a lower VO_2_ signal-to-noise ratio than in heavy and moderate intensity domains. However, other examples are available as supporting information (S1 File). Fluctuations in gas exchange are even more pronounced in swimming given the constraint related to the breathing pattern (e.g., during front crawl there is a specific moment to inspire and other to expire). Unlike swimming, athletes can breathe when they want during running or cycling. Likewise, swallowing and coughing can also generate fluctuations in gas exchange, resulting in variability or ‘noise’ around the mean VO_2_ response [18,32,47]. These errant breaths can degrade the signal quality since they are not components of the response, influencing the confidence in parameter estimates and their interpretation [48]. Thus, the high coefficients of variation of critical estimated parameters also highlight some issues regarding our experimental design.

VO_2_FITTING is also provided with the widely used filters for VO_2_ kinetics analysis, which are described in detail in the supporting information (S1 File). In this regard, we present related data illustrating quantitatively and graphically some of these filters, like *rolling standard deviation*, *averaging in a box* and *moving average*. Other filters like *interpolation every 1-s* and moving mean are also available in VO_2_FITTING (S1 File). Although commonly used, there is little consensus on how to fit and treat swimming VO_2_ kinetics data [10,11]. However, even assuming that errant breaths are not from the actual transient VO_2_ kinetics, editing of the VO_2_ signal should be done with caution using a priori established criteria [29]. As standard values for data editing have not been established yet, some authors prefer to err on the side of less stringency, and exclude data that lie more than four standard deviations away from the mean [12,29,47]. Although symmetrical high–low pairing of breaths may offset fitting effects, it is debatable whether the fitting model should be conducted on filtered or raw data, since more stringency (allowing more ‘errant’ data points) could exert a major influence on the parameters estimation. For example, substantial errors can be observed during fast VO_2_ kinetic responses given the limited volume of data in the transient region [29]. Since model fitting VO_2_ kinetics parameters are necessarily estimates, adequate characterization of its response cannot be satisfactorily retrieved from artificially filtered data where noise is deliberately attenuated [32,49]. Thus, to avoid any constraint, parameter estimates and goodness of fit between different models were only analyzed with raw data.

It is unclear whether time aligning and ensemble averaging VO_2_ data to yield a single transition can affect the physiological interpretation of parameter estimates. Some of the existing modelling methods require subjects to perform several transitions, reducing noise and improving parameter estimates [33,34,35,36]. Although VO_2_FITTING also allows this type of signal processing, a bootstrapping method was chosen to estimate parameters using samples from a unique transition for each participant. This approach provides reliable information about the estimated parameters [37,38,40]. In fact, the estimated coefficients of variation for A_p_ (mono: 1.5% vs. bi-exponential: 5.3%) and TD_p_ (mono: 19% vs. bi-exponential: 16%) in the current study were relatively suitable for both models. However, the low accuracy of the two critical parameters on the bi-exponential model (i.e., A_sc_end_: 35% and TD_sc_: 45%) seems to be related to the pronounced fluctuations in VO_2_ kinetics in swimming, and the inherently low signal-to-noise ratio which typically decreases from childhood through adulthood [23].

Understanding the physiological significance of both VO_2_ fast and slow components during exercise is an essential skill for researchers and performance analysts [21,50,51]. We tested the VO_2_FITTING with data from a T400, usually used to prescribe the target swimming speed for aerobic power development, both in age-group and adult swimmers [16,52]. The workload demand during severe intensity exercise leads to a loss of muscle metabolic homeostasis that compromises the muscle power output, requiring additional motor unit recruitment and increased oxygen cost forming the VO_2sc_ [53]. However, although the bi-exponential model was the best fit for 75% of the current sample when comparing with the mono-exponential model, the sum of squares residuals when fitting this model was smaller for all swimmers [15]. This contradiction may be explained by the inherent breath-by-breath noise observed in young swimmer’s response profiles, which could mask any clear changes in ventilatory variables [23]. Nevertheless, even without significant differences observed between mono- and bi-exponential models for the remaining five swimmers, the mean A_sc_ calculated as the difference between the VO_2_ at the end and the amplitude of the primary phase was ≥2.1 mL·kg^−1^·min^−1^ (Figure 3). These data are suggestive of an imbalance in muscle metabolic homeostasis followed by peripheral fatigue [51,53]. Thus, the current data are consistent with the expected behaviour for exercise response in the severe intensity domain for most of the swimmers who performed the T400 [16,54]. However, since both heavy and severe exercise may evince a slow component [15], concomitant analysis with other physiological variables and swimming techniques could yield a more comprehensive overview of the swimmer profile [55].

Although VO_2_FITTING allows straightforward analysis of respiratory responses for research and performance in sports practitioners, it cannot be considered as the definitive solution for VO_2_ kinetics data processing for novice/regular user because its interpretation requires knowledge about respiratory physiology. Although some commercial software packages provide intuitive graphical interfaces and relatively straightforward options to analyze VO_2_ kinetics data, knowledge about respiratory physiology is also mandatory. Moreover, none of these commercial software packages are free or open-source. VO_2_FITTING offers advantages, but it is important to acknowledge some of the shortcomings and potential limitations of the software. First, although relevant for research and performance assessment, off-transient VO_2_ kinetics analysis is not yet available in this version. However, since VO_2_FITTING is open-source software, other mathematical functions traditionally used to estimate physiological parameters related to VO_2_ kinetics can be incorporated into the software by the authors or contributors. Secondly, technical support and detailed user manuals of VO_2_FITTING (S1 File) are only available in English. Finally, despite the concurrent validity observed for all available models in VO_2_FITTING and examples illustrating its applicability, we have not examined the constraints for parameter estimates, logistic and tri-exponential models. Future challenges include testing of all the remaining available options with experimental data, and updating VO_2_FITTING documentation with examples to illustrate each of these available tools.

## 5. Conclusions

VO_2_FITTING has shown to be valid for characterizing VO_2_ kinetics in exercise. Initial concurrent validation showed perfect fits for all available models, with parameter estimates matching perfectly the known inputted values. The evaluation of severe intensity transitions in swimming has illustrated some applications and feasibilities of VO_2_FITTING. We identified the expected behaviour for severe intensity VO_2_ kinetics for most swimmers, which, if assessed concurrently with other physiological variables and swimming technique analysis should generate a complete (biophysical) overview of a swimmer’s profile [55]. We also showed fitting results when using supplementary swimming and running-related data (S1 File), illustrating other available options of VO_2_FITTING. This freely available software, which analyzes VO_2_ kinetics in exercise, can be applied for research and performance in elite, sub-elite or recreational athletes. Since it is open-source software, we believe that VO_2_FITTING appears to have promise as a useful tool for the sports science community. 

## Figures and Tables

**Table 1:** Estimated VO_2_-related parameters obtained from mono- and bi-exponential models (mean ± SD).

	Mono-Exponential	Bi-Exponential
A_0_ (mL·kg^−1^·min^−1^)	8.8 ± 3.4	8.8 ± 3.4
A_0_ (mL·min^−1^)	528 ± 204	528 ± 204
A_p_ (mL·kg^−1^·min^−1^)	44.1 ± 7.0	40.0 ± 7.3
A_p_ (mL·min^−1^)	2644 ± 419	2398 ± 438
95%CI (mL·kg^−1^·min^−1^)	42.9 to 45.4	35.0 to 42.9
CV (%)	1.5%	5.3%
TD_p_ (s)	20.8 ± 6.1	24.0 ± 6.8
95%CI (s)	13.9 to 26.9	16.5 to 30.4
CV (%)	18.5%	16.3%
τ_p_ (s)	26.5 ± 12.0	16.5 ± 7.4
95%CI (s)	18.3 to 36.4	8.1 to 28.1
CV (%)	16.6%	36.4%
A_sc_end_ (mL·kg^−1^·min^−1^)	-	7.0 ± 1.8
A_sc_end_ (mL·min^−1^)	-	417 ± 108
95%CI (mL·kg^−1^·min^−1^)	-	3.3 to 12.4
CV (%)	-	34.8%
TD_sc_ (s)	-	137 ± 23
95%CI (s)		11 to 240
CV (%)	-	45%
VO_2_ at the end (mL·kg^−1^·min^−1^)	55.1 ± 6.4	55.1 ± 6.4
VO_2_ at the end (mL·min^−1^)	3303 ± 384	3303 ± 384

A_0_ is the VO_2_ at rest; A_p_ and A_sc_end_, TD_p_ and TD_sc_ are respectively amplitudes and corresponding time delays of the fast and slow VO_2_ components. The τ_p_ is the time constant of the fast VO_2_ component. CV (%) and 95%CI are the mean coefficient of variation and 95% confidence interval for each mean parameter estimate, respectively.

## Supplementary Materials

The following are available online at https://www.mdpi.com/2075-4663/7/2/31/s1, **S1 File**: Supporting information 1_VO_2_FITTING Documentation; **S2 File**: Supporting information 2_Validation Datasets.
