# Supplementary material for: VO2FITTING: A Free and Open-Source Software for Modelling Oxygen Uptake Kinetics in Swimming and other Exercise Modalities
Source: Sports (Basel). 2019 Jan 24;7(2):31. doi: 10.3390/sports7020031 (PMC6409559; doi:10.3390/sports7020031)
Supplement: Supplementary file 1 [file sports-07-00031-s001.zip › S1_FILE .docx]

S1 FILE (Supporting information 1): VO_2_FITTING Documentation

ARTICLE: VO_2_FITTING: A free and open-source software for modelling oxygen uptake kinetics in swimming and other exercise modalities

**Rodrigo Zacca ^1,2,3,^ *, Rui Azevedo ^4^, Pedro Figueiredo ^5^, João Paulo Vilas-Boas ^1,2^, Flávio A. de S. Castro ^6^, David B. Pyne ^7^ and Ricardo J. Fernandes ^1,2^**

^1^ Centre of Research, Education, Innovation and Intervention in Sport, Faculty of Sport, University of Porto, Porto, 4200-450 Portugal; jpvb@fade.up.pt (J.P.V.B.), ricfer@fade.up.pt (R.J.F.)

^2^ Porto Biomechanics Laboratory (LABIOMEP), University of Porto, Porto, 4200-450, Portugal

^3^ CAPES Foundation, Ministry of Education of Brazil, Brasília, 70040-031, Brazil

^4^ CESPU, Institute of Research and Advanced Training in Health Sciences and Technologies (IINFACTS), Gandra PRD, 4585-116, Portugal; rui.azevedo@iucs.cespu.pt

^5^ Portugal Football School, Portuguese Football Federation, Cruz Quebrada-Dafundo, 1495-433, Portugal; pedfig@me.com

^6^ Aquatic Sports Research Group, Universidade Federal do Rio Grande do Sul, Porto Alegre, 90690-200, Brazil; souza.castro@ufrgs.br

^7^ Research Institute for Sport and Exercise, University of Canberra, Canberra, ACT 2617, Australia; david.pyne@canberra.edu.au

* Correspondence: [rodrigozacca@yahoo.com.br](mailto:rodrigozacca@yahoo.com.br)

**Contents**

[1. VO_2_FITTING Tool 2](#_Toc532544004)

[2. Installation and quick tutorial 2](#_Toc532544005)

[3. Input 3](#_Toc532544006)

[4. Filtering 8](#_Toc532544007)

[5. Available models 13](#_Toc532544008)

[6. Output 14](#_Toc532544009)

[7. Constraining parameters in curve fitting 18](#_Toc532544014)

[8. Model comparisons 19](#_Toc532544015)

[9. Known issues and future work 19](#_Toc532544016)

[10. References 20](#_Toc532544017)

**1. VO_2_FITTING Tool**

The VO_2_FITTING is a web application based on R language (R Core Team 2015), with support of the “Shiny” [1] package, that provides freely available software for characterizing V̇O_2_ kinetics in exercise. The VO_2_FITTING software provides a dynamic and full analysis of the on-transient V̇O_2_ responses to exercise, offering functionalities that confer enough flexibility to compare simultaneously several cardiodynamic responses with sufficient precision to meet researchers requirements.

**2. Installation and quick tutorial**

To run the software, the following configurations are necessary. VO_2_FITTING runs online inside a browser. Details about the application, source code, installation instructions, and other documentation, can be verified on the landing page (https://shiny.cespu.pt/vo2_news/). Source code is released under a GPL3 license (https://www.r-project.org/Licenses/GPL-3). Likewise, an R environment should be available, where all the app dependencies are installed. The R command line (where VO_2_FITTING folder is located) is needed prior to each launch, using the following commands: *library(shiny)* and *runApp(vo2)*. The following dependencies should be previously installed (Table 1):

| **Table 1.** Required Dependencies | |
| --- | --- |
| Library (shiny) [1] | #Version >= 0.14.2 |
| Library (minpack.lm) [2] | #Version >= 1.2-0 |
| Library (chron) [3] | #Version >= 2.3-47 |
| Library (zoo) [4] | #Version >= 1.7-13 |
| Library (bcrypt) [5] | #Version >= 0.2 |
| Library (rmysql) [6] | #Version >= 0.11-3 |
| Library (digest) [7] | #Version >= 0.6.10 |
| Library (nlstools) [8] | #Version >= 1.0-2 |
| Library (tseries) [9] | #Version >= 0.10-35 |
| Library (openxlsx) [10] | #Version >= 3.0.0 |
| Library (readods) [11] | #Version >= 1.6.4 |

A local *MySQL* or *Mariadb* database server is also necessary. The database needs two tables named “Models” and “Users”, with the following structure, respectively (Table 2 and 3):

| **Table2.** Models | | | | | |
| --- | --- | --- | --- | --- | --- |
| **Field** | **Type** | **Null** | **Key** | **Default** | **Extra** |
| id | mediumint (9) | No | PRI | Null | auto_increment |
| uid | mediumint(9) | No |  | Null |  |
| modeltype | varchar(256) | No |  | Null |  |
| modeldesc | varchar(512) | No |  | Null |  |
| model | varchar(256) | No |  | Null |  |
| data | varchar(256) | No |  | Null |  |
| timestamp | char(25) | No |  | Null |  |

| **Table 3.** Users | | | | | |
| --- | --- | --- | --- | --- | --- |
| **Field** | **Type** | **Null** | **Key** | **Default** | **Extra** |
| id | mediumint(9) | No | PRI | Null | auto_increment |
| username | varchar(60) | Yes | UNI | Null |  |
| password | char(60) | Yes |  | Null |  |

The dump *databasestructure.sql* can be imported if this structure cannot be created manually. Database encoding should be *utf8* and table collation *utf8_general_ci*. The database connection is configured in the file *db.ini*. Currently, there is no web interface to manage users. Thus, the small script *createuser.R* in the command line can be employed to create new users. The access to source code repository is granted upon request by email to the corresponding author.

*Quick tutorial*

1. Click *browse* on the left of *“Upload VO2 data as a function of time”* menu to upload a dataset (read section Input for help);
2. Choose *model* for fitting (or default);
3. Choose *V̇O_2_ baseline* option.

**3. Input**

A text file must be used when setting the default input dataset, following the requirements below (See an example in Figure 1):

1. The first line must have two different columns named by default *t* (time) and *VO2/kg*. They can be located in any position (1^st^ and last columns, for example);
2. The expected time format (default) is hh:mm:ss;
3. The first data point from each column starts (by default) on the 4^th^ line;
4. The expected *VO2/kg* units are mL·kg^-1^·min^-1^;
5. Columns are separated by default with semicolon**;**

However, the following options can be chosen at “Input and Models -> Data format” menu:

1. Other input formats, such as *XLSX* and *ODS*;
2. The sheet name where the data is located;
3. The line where the data starts;
4. The name of the column which contains time data;
5. The name of the column which contains V̇O_2_ values;
6. The time format;
7. The data separator (text file format).

The Input file can have additional columns, which will be ignored. An example of an input dataset spreadsheet is shown in Figure 1.


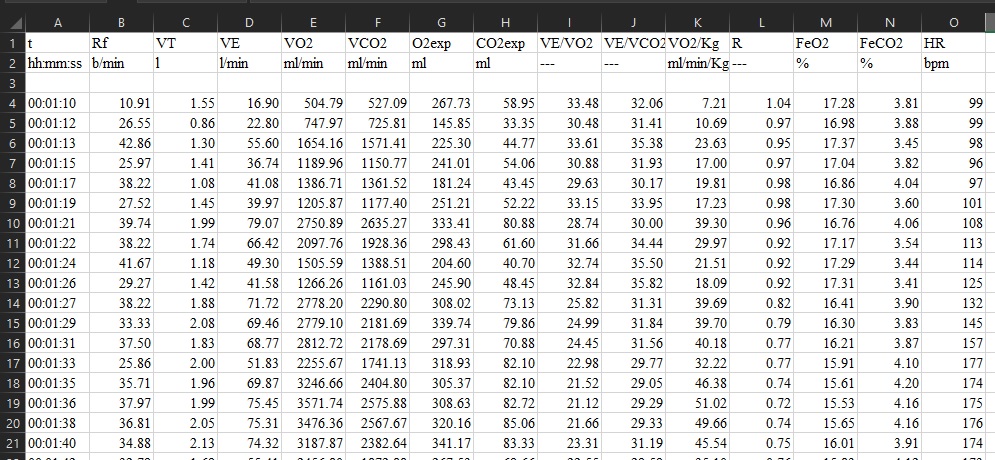


**Figure 1.** Example of input dataset

*Option: multiple files*

If two or more observations need to be time-aligned to yield a single profile, VO_2_FITTING has the option to upload multiple files and combine them in one. It is necessary to hold the *CTRL* button while selecting those files. There are three options to combine the datasets: using median, average or joining all data. By selecting median, if the instant *t=1s* has three observations, VO_2_FITTING will calculate the median for those three observations. The recommended (and the default) option is median, since is less sensitive to outliers. VO_2_FITTING starts by creating a list of all the times when the sampling times are not identical in all the files, as the following example:

If: Times file 1= 1s,3s,7s and Times file 2= 1s,2s,7s; Thus, all Times: 1s,2s,3s,7s

Before combining the V̇O_2_ data, VO_2_FITTING needs information for V̇O_2_ in file 1 for t=2s, and in file 2 for t=3s. VO_2_FITTING uses linear interpolation to find that information. For instance, in the case of V̇O_2_ for file 1 at t=2s, VO_2_FITTING will linearly interpolate from t=1s, to t=3s to estimate what the V̇O_2_ observation would be at t=2s. An example of two time-aligned tests is shown in Figure 2, where one swimmer performed two square wave transitions (5 minutes) at 95% of velocity (vV̇O_2_max) associated with the V̇O_2max_ intensity, separated by a 24 h rest period and performed immediately after ~800-m front crawl warm-up at a moderate intensity. Swimming speed was controlled by a visual pacer with flashing lights at the bottom of the swimming pool (TAR.1.1, GBKelectronics, Aveiro, Portugal).


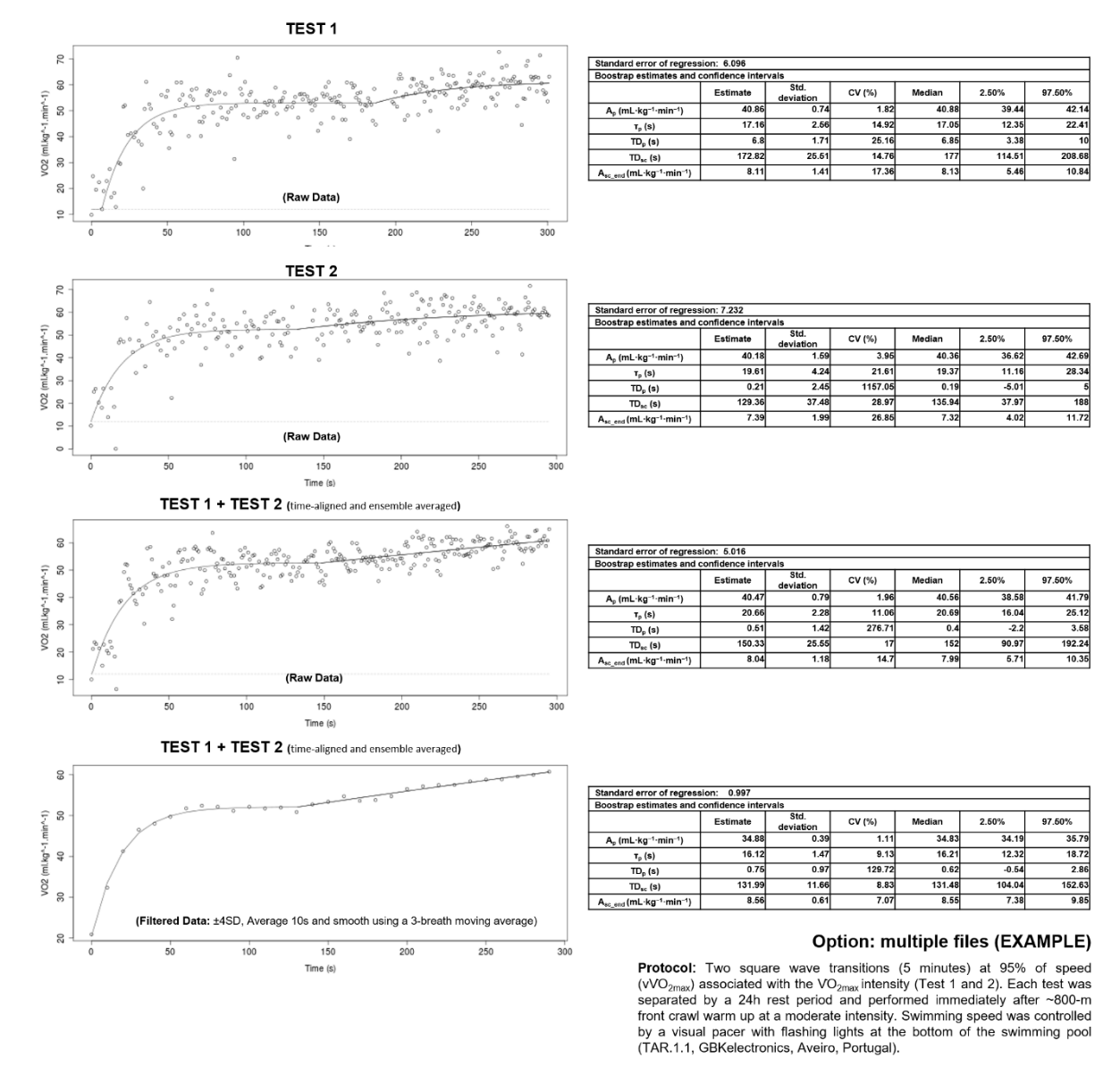


**Figure 2.** Example of two time-aligned tests with *multiple files* option on VO_2_FITTING. From the top to the bottom plots: Test 1 with raw data, Test 2 with raw data, Test 1 and 2 time-aligned with raw data and filtered data.

*Option: click delete points*

This option allows to click on each point, deleting them if necessary. Thus, selected points are removed and not considered for modelling. Soon after, VO_2_FITTING adjusts the fit automatically. By clicking in the same point again or unchecking this option, it is possible to reset it.

*Option:* *show data cutter*

A slider will appear below the plotted graphic when selecting this option. The slider allows to restrict the model fitting in a specific time range.

Figure 3 show examples for *click delete points* and *data cutter* options using a data set obtained from one elite runner during an 7x800-m intermittent protocol performed on a 400-m outdoor running track. The velocity was increased by 1 km·h^-1^ for each 800-m step with a 30-s rest interval until exhaustion and controlled by audio feedback emitted in markers placed at 100-m intervals.


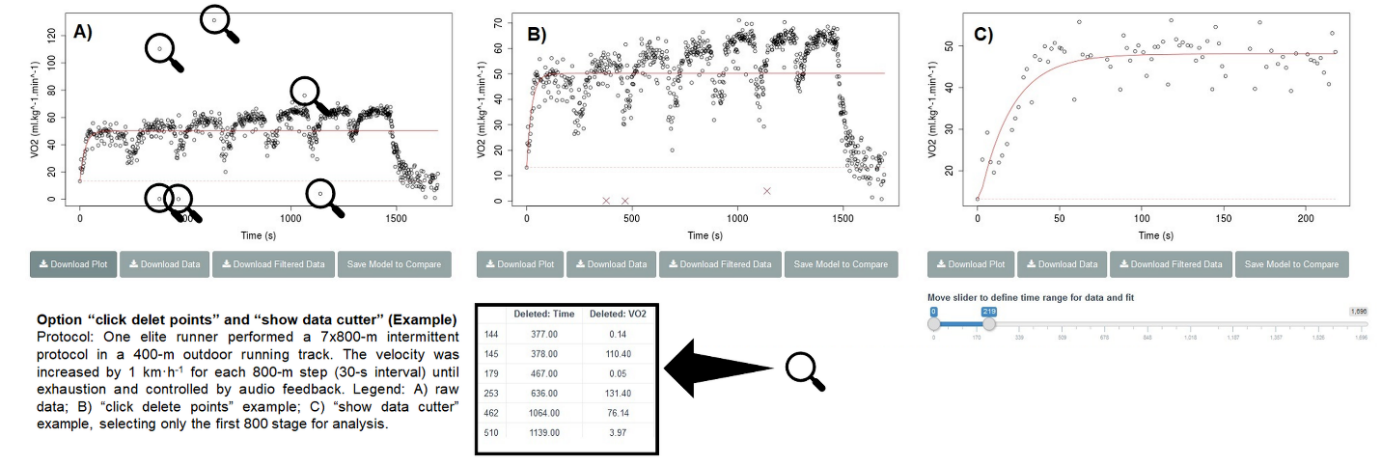


**Figure 3.** Illustration of *click delete points and show data cutter options*. Data from a square wave transition performed in treadmill by an Elite runner.

*Option: multiply by mass*

This option can be used to change V̇O_2_ dataset unit of measurement to ml·min^-1^.

*Option: choose how to define vo_2baseline_*

Adjusted by fitting (manual)

This option will fit the V̇O_2baseline_ automatically, limiting the lower/upper range of the parameter for fitting to manually set values. Starting value will be calculated as the middle of the range.

Adjusted by fitting (automatic)

This option can be used to fit V̇O_2baseline_ automatically, ranging from 0 to +Inf, with a starting value of 40 (not really meaningful).

Manual Value

By selecting this option, the V̇O_2baseline_ can be introduced manually, usually in units of mL·kg^-1^·min^-1^. If the “Multiply by mass” option has been chosen, then V̇O_2baseline_ in mL·min^−1^ can be introduced.

Manual Value backwards repetition

This option can be useful if input data has V̇O_2_ values that starts from the beginning of the exercise (t=0), but V̇O_2baseline_ is in another data file. This is one option to fit the onset of the first exponential by creating artificial baseline data, so the time delay can be accurately determined.

Average/Median of first X points

By setting this option, there is no limit for the number of points to define V̇O_2baseline._

First data point

By selecting this option, the first V̇O_2_ data point can be defined as the V̇O_2baseline_ value.

**4. Filtering**

It is arguable whether the model fitting should be conducted with filtered or raw data, since more stringency (allowing more 'errant' data points) could exert a major influence on parameter estimation. However, all filters mentioned below are available on VO_2_FITTING and can be applied before fitting the model and by the order which are selected.

*Moving average*

Simple moving average using R’s filter function. The number of points when using this option can be specified in the interface. An odd number of points is recommended.

*Moving median*

Simple moving median using R’s runmed function. The number of points when using this option can be specified in the interface. An odd number of points is required.

*Interpolate every 1s*

This filter will fill gaps using linear interpolation every 1s. For example, in one file with data points: (t=1s, V̇O_2_=30) (t=4s, V̇O_2_=40), VO_2_FITTING will create new data points at t=2s and t=3s.

*Averaging in a box*

By selecting this filter, VO_2_FITTING will create boxes of the specified number of seconds and average all the data in that box.

For example, consider the data:

- t=101s, V̇O_2_=30
- t=102s, V̇O_2_=40
- t=103s, V̇O_2_=50
- t=104s, V̇O_2_=60

By selecting *averaging in a box* is a box of 2 seconds, the new data will be:

- t=101.5s, V̇O_2_=35
- t=103.5s, V̇O_2_=55

(Note: this exact behavior is still being worked on.)

*Rolling standard deviation*

Using an odd number of points, this filter calculates rolling mean and rolling standard deviation, excluding points which are above or below local mean ± threshold×std. deviation. For better results, an option which calculates the median of all rolling std. deviations is available an active by default. This median is then used as the reference std. deviation, rather than the local std. deviation. The number of points and the threshold can be chosen, which by default is 4. The exclusion of these aberrant values (> 4 standard deviations about the local mean) of V̇O_2_ is justified by the fact that they typically arise due to swallowing or coughing, or some other reason unrelated to the physiological response of interest [12, 14]. It is important to note that for a width of 3, one point is removed in the beginning and in the end. For a width of 5, two points are removed and so on. Data with few points in the beginning might be a problem for fitting V̇O_2_ kinetics.

Table 4, Table 5 and Figure 4 illustrate quantitatively and graphically the use of selected filters in different mathematical models with datasets from swimming (n=1; adult) and running (N=1; adult), particularly V̇O_2_ related parameters and fits obtained from mono- and bi-exponential models (raw and filtered data) during square wave transitions at 100% of speed (vV̇O_2max_) associated with the V̇O_2max_ intensity.

| **Table 4.** Estimated V̇O_2_ related parameters obtained from a mono-exponential model from running (n=1) and swimming (n=1) during a square wave transition at 100% of vV̇O_2max_, using raw and filtered data (±4SD, average 10s and smooth by a 3-breath moving average) | | | | |
| --- | --- | --- | --- | --- |
|  | **Raw** | | **Filtered** | |
|  | **Mono-exponential**  **Swimming (N=1)** | **Mono-exponential**  **Running (N=1)** | **Mono-exponential**  **Swimming (N=1)** | **Mono-exponential**  **Running (N=1)** |
| A_0_ (mL·kg^−1^·min^−1^) | 10.2 | 8.8 | 10.2 | 8.8 |
| A_p_ (mL·kg^−1^·min^−1^) | 47.3 | 55.1 | 46.84 | 55.2 |
| CV (%) | 1.4% | 0.6% | 0.8% | 0.8% |
| TD_p_ (s) | 20.9 | 23.6 | 17.8 | 18.0 |
| CV (%) | 12.0% | 2.6% | 4.3% | 2.2% |
| τ_p_ (s) | 32.9 | 15.1 | 30.4 | 15.8 |
| CV (%) | 12.3% | 6.4% | 5.6% | 7.0% |
| A_sc_end_ (mL·kg^−1^·min^−1^) | - | - | - | - |
| CV (%) | - | - | - | - |
| TD_sc_ (s) | - | - | - | - |
| CV (%) | - | - | - | - |
| τ_sc_ (s) | - | - | - | - |
| CV (%) | - | - | - | - |
| V̇O_2_ at the end (mL·kg^−1^·min^−1^) | 58.8 | 67.2 | 57.4 | 66.7 |
| vV̇O_2_ max (m.s^-1^) | 1.38 | 5.10 | 1.38 | 5.10 |
| Time limit at vV̇O_2_ max (s) | 259 | 220 | 259 | 220 |

A_0_ is the V̇O_2_ at rest; A_p_ and A_sc_end_, TD_p_ and TD_sc_, and τ_p_ and τ_sc_ are respectively amplitudes, corresponding time delays and time constants of the fast and slow V̇O_2_ components. CV (%) is the coefficient of variation for each parameter estimate.

| **Table 5.** Estimated V̇O_2_ related parameters obtained from a bi-exponential model from running (n=1) and swimming (n=1) during a square wave transition at 100% of vV̇O_2max_, using raw and filtered data (±4SD, average 10s and smooth by a 3-breath moving average) | | | | |
| --- | --- | --- | --- | --- |
|  | **Raw** | | **Filtered** | |
|  | **Bi-exponential**  **Swimming (N=1)** | **Bi-exponential**  **Running (N=1)** | **Bi-exponential**  **Swimming (N=1)** | **Bi-exponential**  **Running (N=1)** |
| A_0_ (mL·kg^−1^·min^−1^) | 10.2 | 8.8 | 10.2 | 8.8 |
| A_p_ (mL·kg^−1^·min^−1^) | 43.4 | 50.0 | 42.7 | 50.2 |
| CV (%) | 4.7% | 3.6% | 8.9% | 2.5% |
| TD_p_ (s) | 29.8 | 26.1 | 18.2 | 18.52 |
| CV (%) | 10.3% | 2.5% | 4.9% | 0.8% |
| τ_p_ (s) | 17.7 | 8.9 | 26.0 | 11.2 |
| CV (%) | 26.2% | 13.6% | 18.6% | 6.0% |
| A_sc_end_ (mL·kg^−1^·min^−1^) | 5.9 | 9.4 | 5.3 | 8.6 |
| CV (%) | 39.7% | 16.44% | 72.5% | 16.6% |
| TD_sc_ (s) | 131.7 | 82.9 | 81.4 | 81.9 |
| CV (%) | 55.8% | 29.18% | 86.9% | 27.9% |
| τ_sc_ (s) | 10108 | 29854 | 17498 | 6587 |
| CV (%) | 765% | 460% | 713% | 995% |
| V̇O_2_ at the end (mL·kg^−1^·min^−1^) | 58.8 | 67.2 | 57.4 | 66.7 |
| vV̇O_2_ max (m.s^-1^) | 1.38 | 5.10 | 1.38 | 5.10 |
| Time limit at vV̇O_2_ max (s) | 259 | 220 | 259 | 220 |

A_0_ is the V̇O_2_ at rest; A_p_ and A_sc_end_, TD_p_ and TD_sc_, and τ_p_ and τ_sc_ are respectively amplitudes, corresponding time delays and time constants of the fast and slow V̇O_2_ components. CV (%) is the coefficient of variation for each parameter estimate.


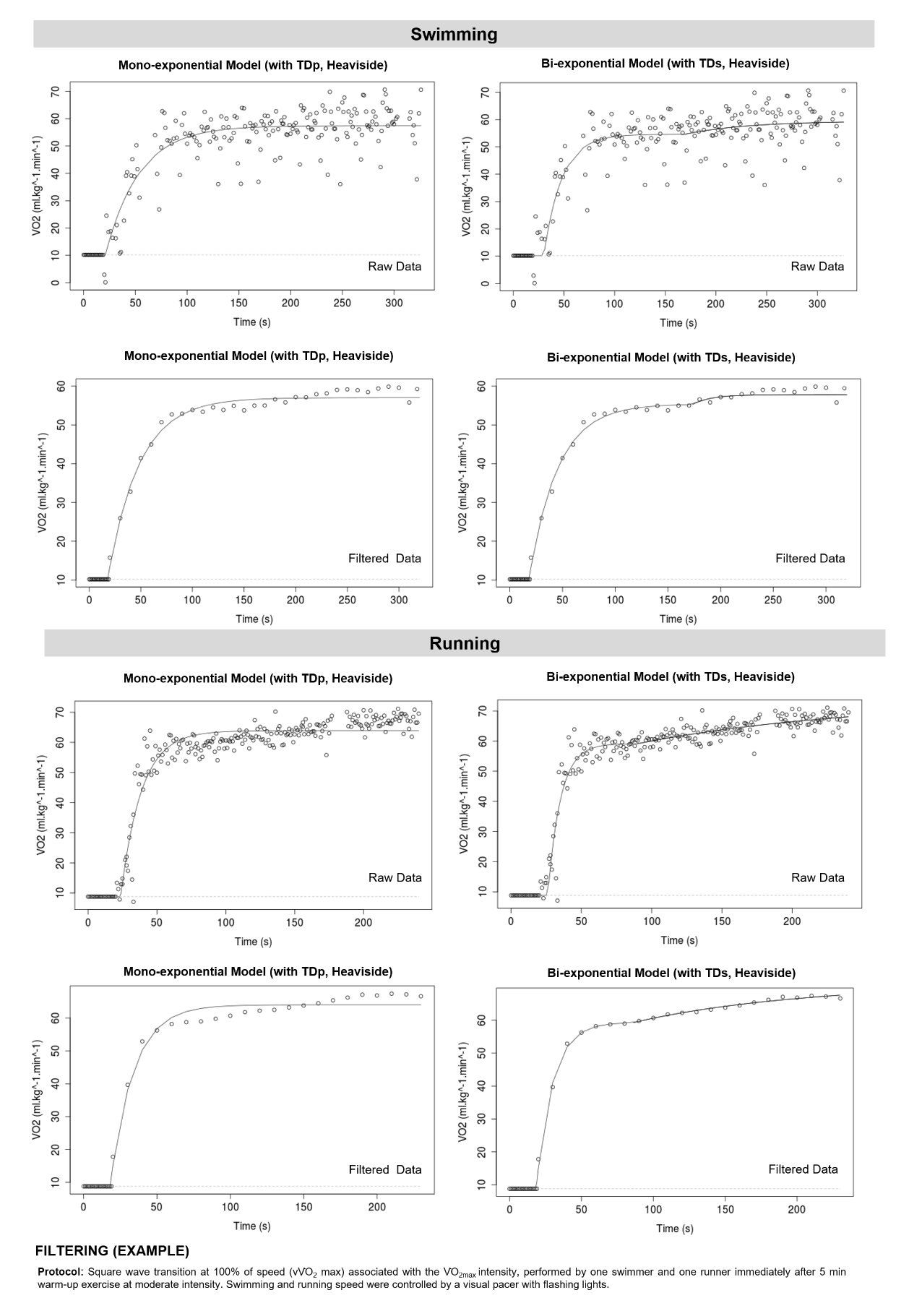


**Figure 4.** Example of V̇O_2_ fits obtained from a mono- and bi-exponential models from running (n=1) and swimming (n=1) during a square wave transition at 100% of vV̇O_2max_, using raw and filtered data (±4SD, average 10s and smooth by a 3-breath moving average)

**5. Available models**

Find below the available models:

*Mono-exponential (no TDp)*

There is no time delay in this model, so it is assumed that the exponential starts nearly or on the first data point.

$$\dot{\text{V}}\text{O}_{\text{2baseline}}\text{+}\text{A}_{\text{P}}\left( \text{1-}\text{e}^{\text{-}\frac{\text{t}}{\text{τ}_{\text{p}}}} \right)$$

*Mono-exponential (with TDp, Heaviside)*

The first 20 s of data after the onset of exercise (cardiodynamic phase) is not considered for V̇O_2_ kinetics analysis in this model. This model also includes the Heaviside step function [13].

Given the Heaviside step function:

$$H\left( \text{t} \right)\text{=}\left\{ \begin{aligned} \text{0,t<0} \\ \text{1,t≥0} \end{aligned} \right.$$

The model consists of a mono-exponential, with the onset only after TDp (Time delay of primary phase):

$$\dot{\text{V}}\text{O}_{\text{2baseline}}\text{ +H(t-}\text{TD}_{\text{p}}\text{)}\text{A}_{\text{P}}\left( \text{1-}\text{e}^{\text{-}\frac{\text{t-}\text{TD}_{\text{p}}}{\text{τ}_{\text{p}}}} \right)$$

*Bi-exponential (with TDs, Heaviside)*

This is bi-exponential model with flexible time delays for the onset of each exponential. The first 20-s of data after the onset of exercise (cardiodynamic phase) are not considered for V̇O_2_ kinetics analysis in this model. This model also includes the Heaviside step function for both exponentials [13]. The model consists of two exponentials, where the first one only starts effectively after TDp (time delay of primay phase) and the second after TDsc (time delay of slow component):

$$\dot{\text{V}}\text{O}_{\text{2baseline}}\text{ +H(t-}\text{TD}_{\text{p}}\text{)}\text{A}_{\text{P}}\left( \text{1-}\text{e}^{\text{-}\frac{\text{t-}\text{TD}_{\text{p}}}{\text{τ}_{\text{p}}}} \right)\text{+H(t-}\text{TD}_{\text{sc}}\text{)}\text{A}_{\text{sc}}\left( \text{1-}\text{e}^{\text{-}\frac{\text{t-}\text{TD}_{\text{sc}}}{\text{τ}_{\text{sc}}}} \right)$$

*Bi-exponential (no TDp, Heaviside)*

The model consists of two exponentials, the first one starts immediately, and the other after TDsc. This model includes Heaviside step functions [13] for the second exponential:

$$\dot{\text{V}}\text{O}_{\text{2baseline}}\text{ +}\text{A}_{\text{P}}\left( \text{1-}\text{e}^{\text{-}\frac{\text{t}}{\text{τ}_{\text{p}}}} \right)\text{+H(t-}\text{TD}_{\text{sc}}\text{)}\text{A}_{\text{sc}}\left( \text{1-}\text{e}^{\text{-}\frac{\text{t-}\text{TD}_{\text{sc}}}{\text{τ}_{\text{sc}}}} \right)$$

*Mono-exponential and linear slow comp. (Heaviside)*

This model is similar to previous one (Bi-exponential, with TDs, Heaviside), but rather than considering the onset of a second exponential, the onset of a linear function is considered:

$$\dot{\text{V}}\text{O}_{\text{2baseline}}\text{ +H(t-}\text{TD}_{\text{p}}\text{)}\text{A}_{\text{P}}\left( \text{1-}\text{e}^{\text{-}\frac{\text{t-}\text{TD}_{\text{p}}}{\text{τ}_{\text{p}}}} \right)\text{+H(t-}\text{TD}_{\text{sc}}\text{)}\text{τ}_{\text{sc}}\left( \text{t-}\text{TD}_{\text{sc}} \right)$$

*Logistic Model*

This experimental model was included for those situations where V̇O_2_ profile is similar to a logistic function:

$$\dot{\text{V}}\text{O}_{\text{2baseline}}\text{ +(}\text{A}_{\text{P}}\text{-}\dot{\text{V}}\text{O}_{\text{2baseline}}\text{)/}\left( \text{1+}\text{e}^{\frac{\text{T}_{\text{mid}}\text{-t}}{\text{τ}_{\text{p}}}} \right)$$

*Tri-exponential (Heaviside)*

Although there are a few studies using tri-exponential models, it is also available, with onsets at TDcd (cardiodynamic phase), TDp and TDsc:

$$\dot{\text{V}}\text{O}_{\text{2baseline}}\text{+H(t-}\text{TD}_{\text{cd}}\text{)}\text{A}_{\text{cd}}\left( \text{1-}\text{e}^{\text{-}\frac{\text{t-}\text{TD}_{\text{cd}}}{\text{τ}_{\text{cd}}}} \right)\text{+H(t-}\text{TD}_{\text{p}}\text{)}\text{A}_{\text{P}}\left( \text{1-}\text{e}^{\text{-}\frac{\text{t-}\text{TD}_{\text{p}}}{\text{τ}_{\text{p}}}} \right)\text{+H(t-}\text{TD}_{\text{sc}}\text{)}\text{A}_{\text{sc}}\left( \text{1-}\text{e}^{\text{-}\frac{\text{t-}\text{TD}_{\text{sc}}}{\text{τ}_{\text{sc}}}} \right)$$

**6. Output**

*Main output window*

Plot of data and fit

- - By clicking anywhere in the plot, the coordinates of the nearest time (s) and V̇O_2_ data point appear.
  - By clicking *download Data* or “Download Filtered Data”, time (s) and V̇O_2_ in *CSV* format can be downloaded, with all the remaining columns might ignored. The *ID of the study* (defined in the interface) determines the name of the file. *Description of the study* will be affixed to the end of the file.
  - By clicking *Save Model to Compare*, after logging in, a dialog will appear asking for a small description of the model to make it easier to identify it later while comparing models in the “Input and models -> Show saved models” section. After clicking *OK* the data and the current model are saved in the database together with the small description.


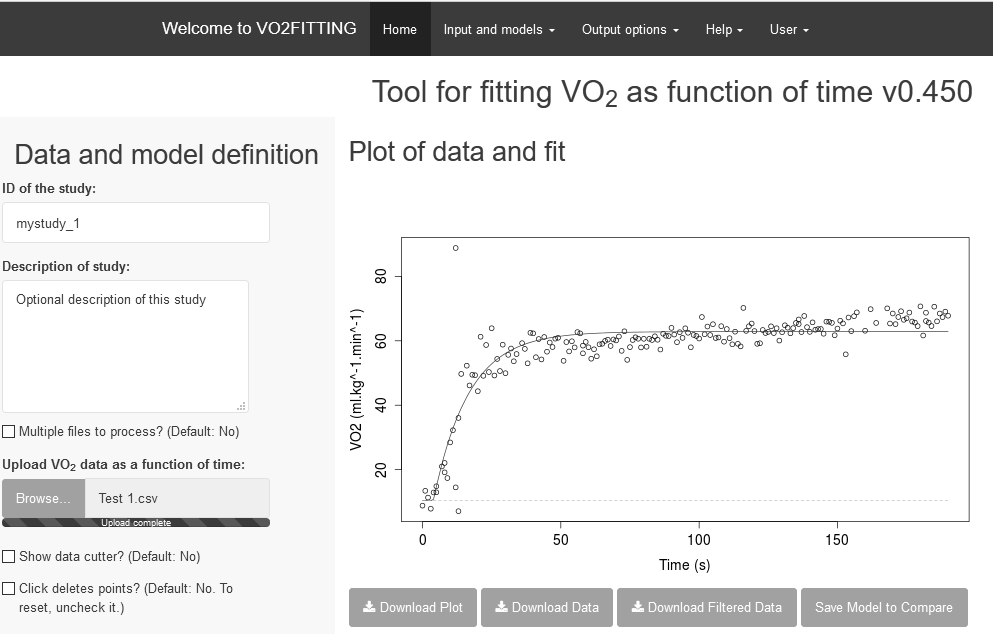


**Figure 5.** *Main output* window *(*Plot of data and fit)

Fitting Results

- - The first table(s) list(s) shows: estimate of the fitted parameters, standard error, t-value and a two-sided p-value for the null/alternative hypothesis “H0 - the parameter is zero/Ha - the parameter is significantly different from zero”, i.e., “this parameter contributes to the explanatory power of the model” (if p-value less than the significance level, defined a priori). In *Output Options -> Auxiliary Reports* shows confidence intervals for the parameter estimates using profile likelihood (stable if data is not noisy). For details on the calculations of these quantities, mostly based on stats and nlstools packages, check comments in the files server.R and helpers.R.
  - V̇O_2baseline_ and mean V̇O_2_ data in the last 30 and 60 seconds (using raw and filtered data) are shown next to this table;
  - By selecting the option “*Show bootstrap confidence intervals*” in *Output Options -> Auxiliary reports,* bootstrap estimates of the parameters will be calculated (This option can slow the application). There is no limit for the number of bootstrap samples (by default: 1000). The routine is based on the code from *nlstools* package [15].
  - By selecting the option *Show a table of the 5 points where V̇O_2_ is higher* in *Output Options -> Auxiliary reports*, a table of the 5 points where the V̇O_2_ is higher (with and without subtraction of V̇O_2baseline_) with raw and filtered data is shown.
  - By selecting the option *Show: V̇O_2_ mean (30s and 60s) - Ap* in *Output Options -> Auxiliary reports*, a table of the average of the V̇O_2_ in the last 30s and 60s with the fitted fast component amplitude (A_p_) subtracted is also shown, using raw and filtered data. This option is useful for some intensity domains where the asymptotic value of the second function is not necessarily reached at the end of the exercise.
  - By selecting the option *Calculate and show CV for the last 30s and 60s* in *Output options -> Auxiliary reports*, a table with the coefficients of variation for the last 30s and 60s of the data is also shown (raw and filtered data).
  - By selecting the option *Calculate and show slow component rigid intervals*, a table of the mean of V̇O_2_ in the end of the data (the number of seconds to average out can be specified) less the average of V̇O_2_ at specific time intervals is shown, as well as the mean of those differences and standard deviations. When: (1) upload data with initial rest; (2) choosing *V̇O_2baseline_ manual value with backwards repetition*”; it is possible to define where the exercise starts in seconds for the rigid intervals to make sense. Thus, when, for example 100 to 120 s in the output, it means time in seconds. Note: it is possible to input negative values.
    - V̇O_2_end- V̇O_2_ (100_to_120) s;
    - V̇O_2_end- V̇O_2_ (90_to_120) s;
    - V̇O_2_end- V̇O_2_ (80_to_120) s;
    - V̇O_2_end- V̇O_2_ (lower and upper limits you may define in the interface) s.

Plots of residuals

The plots of residuals permits evaluation of the goodness-of-fit of the model. Several plots are shown by default:

- - Standardized residuals vs time;
  - Residuals vs time;
  - Standardized residuals vs fitted values;
  - Partial autocorrelation function as a function of lag;
  - Histogram of residuals with a Gaussian distribution on top of it;

The following statistical analysis are available after the plots of the residuals:

- - *the output of a Shapiro-Wilk test on the residuals.* If the p-value of the test is below the significance level that was defined a priori, it is an indication that the residuals are probably not normally distributed.
  - *the simple run test output.* If the p-value of the test is below the significance level that was defined a priori, it is an indication that the residuals are probably autocorrelated.

Figure. 6 shows a screenshot from the bottom of the VO_2_FITTING home menu detailing residuals plots to evaluate the goodness of fit of the T400 modelled (swimming) V̇O_2_ response (bi-exponential) of the same swimmer presented in Fig 2 of the main manuscript.


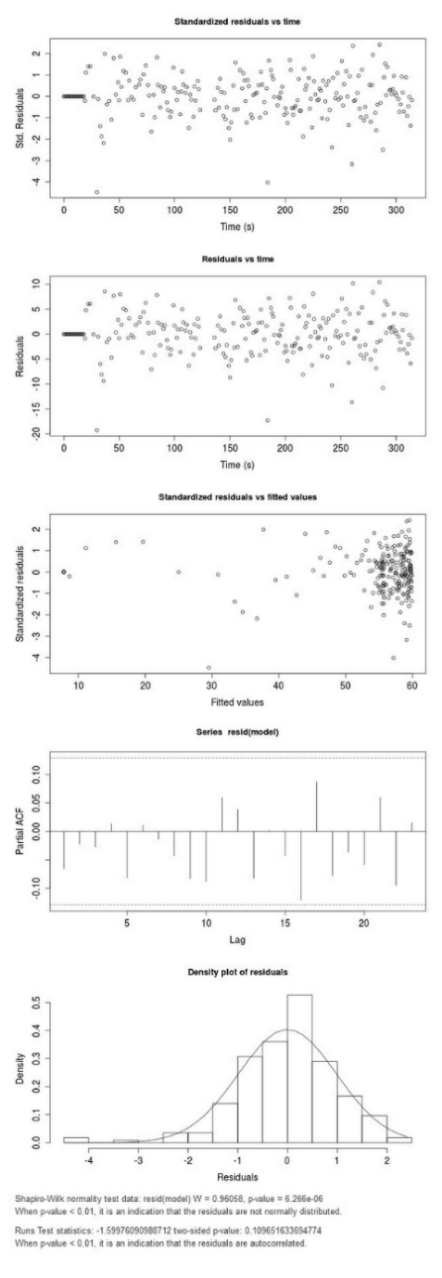


**Figure. 6** Residuals plots from a T400 (swimming) modelled V̇O_2_ response (bi-exponential). From the top to the bottom: *Standardized residuals* and *residuals vs time*, *standardized residuals vs fitted values*, partial autocorrelation function as a function of lag, histogram of residuals with a Gaussian distribution, output of a Shapiro-Wilk test on the residuals and the output of a simple test are shown.

Confidence Regions - Contours based on RSS

- - By selecting the option *Show contours based on the residual sum of squares* in *Output Options -> Auxiliary reports*, contours based on RSS will be available (this option can also slow the application). Expansion factor of the parameter intervals defining the grids can be selected. The factor can be increased if the 95 percent Beale’s confidence regions exceed the plot size and a better view is desired. The routine is based on the code from *nlstools* package [15].

**7. Constraining parameters in curve fitting**

There is a functionality which allows individual constraining parameters (to setting the limits). These parameter procedures are located in the home menu, as shown in Figure 7:


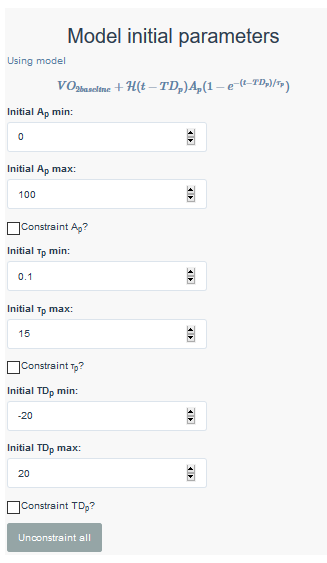


Figure 7: Constraining parameters window for Mono-exponential with TDp, Heaviside) curve fitting.

**8. Model comparisons**

By selecting the option *Show Saved Models* in *Input Models -> Show Saved Models*, a list of saved fits is shown. By selecting the models to compare and clicking in *Compare Models*, a plot with the fit of all compared models will be shown (see Figure 8). It is important to observe the order which each model is selected. The first selected model defines the corresponding base data points to be shown. A legend is automatically added. To better understand the functions used in the modelling, check the beginning of the file helpers.R. It is possible to select up to 21 saved models to compare at the same time, or only one for individual visual analysis.


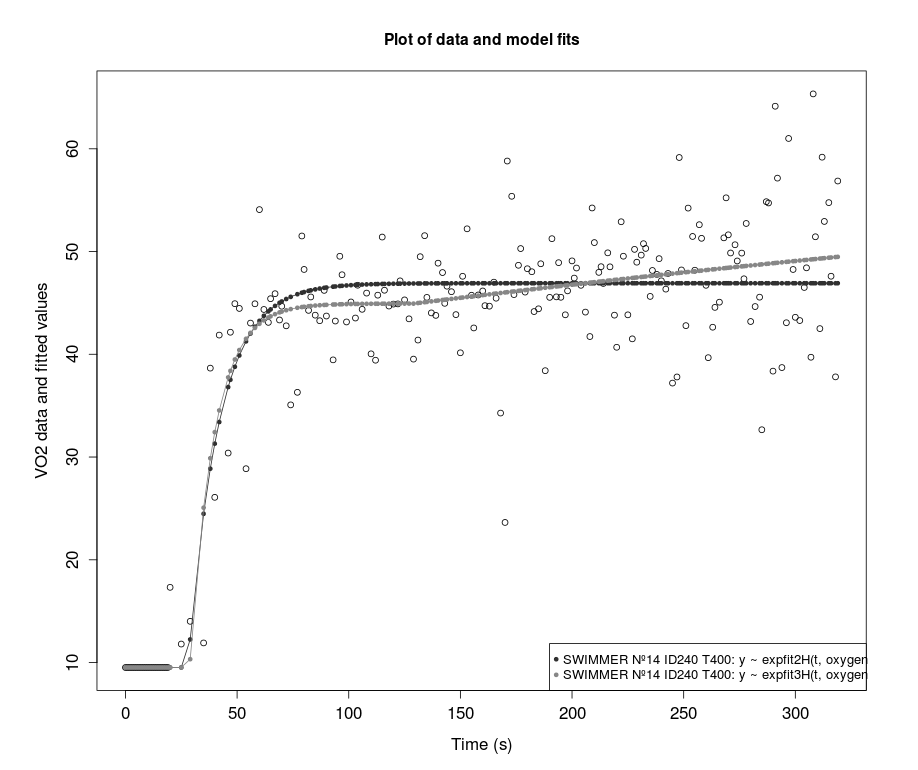


**Figure 8.** Example of model comparison between two saved fits.

The currently available option to compare each model is ANOVA, in which comparison analysis will be displayed above the plot. It is up to the user to check if the analysis makes sense or not, which will depend on the models chosen for comparison.

**9. Known issues and future work**

<https://gitlab.com/vo2fitting/app/issues>

# **10. References**

1. Chang W, Cheng J, Allaire J, Xie Y, McPherson J. Shiny: web application framework for R. R package version 0.11. 2015;1. http://CRAN.R-project.org/package=shiny
2. Elzhov TV, Mullen KM, Bolker B. R interface to the Levenberg-Marquardt nonlinear least-squares algorithm found in MINPACK. Plus Support for Bounds. 2010. https://CRAN.R-project.org/package=minpack.lm.
3. James D, Hornik K, Grothendieck G. chron: Chronological objects which can handle dates and times. R package version. 2013;23:4-4.http://CRAN.R-project.org/package=chron.
4. Zeileis A, Grothendieck G. zoo: S3 infrastructure for regular and irregular time series. arXiv preprint math/0505527. 2005 May 25. doi:10.18637/jss.v014.i06
5. Ooms, Jeroen. 2015. “Bcrypt: ’Blowfish’ Password Hashing Algorithm.” https://CRAN.R-project.org/package=bcrypt.
6. Ooms J, James D, DebRoy S, Wickham H, Horner J. RMySQL: database interface and MySQL driver for R. R package version 0.10. 2015;3.
7. Antoine Lucas, Dirk Eddelbuettel with contributions by, Jarek Tuszynski, Henrik Bengtsson, Simon Urbanek, Mario Frasca, Bryan Lewis, Murray Stokely, et al. 2016. “Digest: Create Compact Hash Digests of R Objects.” https://CRAN.R-project.org/package=digest.
8. Baty F, Ritz C, Charles S, Brutsche M, Flandrois JP, Delignette-Muller ML. A toolbox for nonlinear regression in R: the package nlstools. Journal of Statistical Software. 2015 Aug 27;66(5):1-21.
9. Trapletti A, Hornik K, LeBaron B. Tseries: time series analysis and computational finance. R package version 0.10-11. 2007. http://CRAN.R-project.org/package=tseries.
10. Walker A. openxlsx: Read, Write and Edit XLSX Files, 2015. R package version.;2(0). https://CRAN.R-project.org/package=openxlsx.
11. Schutten, Gerrit-Jan, Chung-hong Chan, and Thomas J. Leeper. 2016. “ReadODS: Read and Write Ods Files.” https://CRAN.R-project.org/package=readODS.
12. Lamarra, N., B. J. Whipp, S. A. Ward, and K. Wasserman. 1987. “Effect of interbreath fluctuations on characterizing exercise gas exchange kinetics.” J. Appl. Physiol. 62 (5): 2003–12.
13. Ma S, Rossiter HB, Barstow TJ, Casaburi R, Porszasz J. Clarifying the equation for modeling of V̇o 2 kinetics above the lactate threshold. Journal of Applied Physiology. 2010 Oct 1;109(4):1283-4. doi:10.1152/japplphysiol.00459.2010.
14. Özyener F, Rossiter HB, Ward SA, Whipp BJ. Influence of exercise intensity on the on‐and off‐transient kinetics of pulmonary oxygen uptake in humans. The Journal of Physiology. 2001 Jun 1;533(3):891-902. doi:10.1111/j.1469-7793.2001.t01-1-00891.x
15. R Core Team. 2015. “R: A Language and Environment for Statistical Computing.” Vienna, Austria: R Foundation for Statistical Computing. https://www.R-project.org/

| 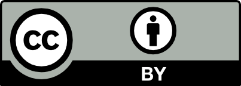 | © 2018 by the authors. Submitted for possible open access publication under the terms and conditions of the Creative Commons Attribution (CC BY) license (http://creativecommons.org/licenses/by/4.0/). |
| --- | --- |
